# Spray Freeze Dried Lyospheres^®^ for Nasal Administration of Insulin

**DOI:** 10.3390/pharmaceutics13060852

**Published:** 2021-06-08

**Authors:** Tuğrul Mert Serim, Jan Kožák, Annika Rautenberg, Ayşe Nurten Özdemir, Yann Pellequer, Alf Lamprecht

**Affiliations:** 1 Department of Pharmaceutics, Institute of Pharmacy, University of Bonn, 53121 Bonn, Germany; mertserim@gmail.com (T.M.S.); s6jakoza@uni-bonn.de (J.K.); annika.rautenberg@uni-bonn.de (A.R.); 2 Department of Pharmaceutical Technology, Faculty of Pharmacy, Ankara University, 06560 Ankara, Turkey; Nurten.Ozdemir@pharmacy.ankara.edu.tr; 3 PEPITE (EA4267), University of Burgundy/Franche-Comté, 25030 Besançon, France; yann.pellequer@univ-fcomte.fr

**Keywords:** spray freeze drying, lyophilization, nasal drug delivery, peptide formulations, porous particles, pharmacokinetic

## Abstract

Pharmacologically active macromolecules, such as peptides, are still a major challenge in terms of designing a delivery system for their transport across absorption barriers and at the same time provide sufficiently high long-term stability. Spray freeze dried (SFD) lyospheres^®^ are proposed here as an alternative for the preparation of fast dissolving porous particles for nasal administration of insulin. Insulin solutions containing mannitol and polyvinylpyrrolidone complemented with permeation enhancing excipients (sodium taurocholate or cyclodextrins) were sprayed into a cooled spray tower, followed by vacuum freeze drying. Final porous particles were highly spherical and mean diameters ranged from 190 to 250 µm, depending on the excipient composition. Based on the low density, lyospheres resulted in a nasal deposition rates of 90% or higher. When tested in vivo for their glycemic potential in rats, an insulin-taurocholate combination revealed a nasal bioavailability of insulin of 7.0 ± 2.8%. A complementary study with fluorescently labeled-dextrans of various molecular weights confirmed these observations, leading to nasal absorption ranging from 0.7 ± 0.3% (70 kDa) to 10.0 ± 3.1% (4 kDa). The low density facilitated nasal administration in general, while the high porosity ensured immediate dissolution of the particles. Additionally, due to their stability, lyospheres provide an extremely promising platform for nasal peptide delivery.

## 1. Introduction

The therapeutic use of biologicals has been gaining increasing importance and with the increasing number of such complex molecules like peptides, proteins, and antibodies, related delivery approaches become of major importance. However, they require parenteral administration, which is in case of a long-term/lifelong therapy accompanied with low patient compliance and with further complications caused by the repetitive invasive administration (e.g., infections, thrombosis). Nasal route has been found to be a promising non-invasive delivery route for peptide drugs as the permeability of the epithelial barrier permits a significant transport of such compounds [1]. Besides, this is chaperoned by the extensive vascularization of the nasal cavity [2] so that the use of liquid dosage forms can be suitable for delivery via the nasal route [3,4].

Liquid formulation-based sprays are currently in use for nasal administration of peptide drugs, however, aqueous solutions of biologicals always risk the general drawback of a limited stability during storage. Accordingly, dry powder formulations have been suggested to overcome this issue [5]. However, the conventional drying techniques such as spray-drying can compromise the stability of sensitive drugs (e.g., peptides and proteins) [6,7,8]. Although the nasal route is a promising non-invasive route for the delivery of peptides and proteins, it shows a high variation in efficacy due to changes in residence time in the nasal cavity depending on the particle properties [9]. Rapid dissolution is essential to prevent clearance of the particles from the mucosa and hence for sufficient bioavailability as well as rapid onset of action. In case of a delayed dissolution of particles on the mucus, this can directly impact on the success of the therapy. Therefore, fast-dissolving, preferentially porous hydrophilic particles, could circumvent this effect and be a prominent alternative for such an application. 

Spray freeze-dried particles are proposed here to offer a solution to the above-mentioned problematics. Generally, spray freeze drying (SFD) is a process in which a solution is dispersed into a freezing medium and dried by sublimation, resulting in lyophilized powders of high porosity, preferentially with spherically shaped particles [10,11,12]. Techniques differ in terms of nozzle type, freezing step, and drying procedure. Omitting the direct contact of the sprayed droplets with the cryogenic liquid facilitated the process handling in many ways and is nowadays used in many cases [13]. The gentle processing of the active in this technique renders the drying of thermo-labile substances possible at low temperatures to produce peptide and protein formulations without activity loss of the biologicals [10]. Besides, specifically, the nasal administration of lyophilisates would have the significant benefit of rapid dissolution of the drug loaded particle on the mucosal surface.

However, earlier studies mainly focused on the delivery of vaccines where amounts of the active and particle masses that need to be administered are distinctly lower in volume [14,15]. Accordingly, we have tested insulin which is of limited structural stability in an aqueous formulation [16,17] and during the spray-drying process [7] and typically needs to be administered in larger amounts to exercise its therapeutic effect. These characteristics make insulin a good model drug for testing the general applicability of the lyospheres for nasal administration of biologicals. 

In this study, the potential of spray-freeze dried lyospheres for insulin delivery was investigated in view of achieving a therapeutically relevant nasal bioavailability of insulin by testing different permeation enhancers in a rat model. In addition, the general validity of these findings was analyzed by testing nasal absorption of fluorescently labeled dextrans of various molecular weights (4 to 70 kDa) from lyospheres in view of the hypothesis that also larger biomolecules than insulins could be delivered in the same way. 

## 2. Materials and Methods

### 2.1. Materials 

Recombinant human insulin was purchased from Merck (Darmstadt, Germany). D(-)-Mannitol was obtained from VWR International (Amsterdam, The Netherlands). Polyvinylpyrrolidone (Kollidon^®^ 12 PF) was a kind gift from BASF (Ludwigshafen, Germany). Sodium taurocholate, β-cyclodextrin, low molecular weight chitosan, fluorescein sodium, fluorescein isothiocyanate, and FITC-labeled dextrans were purchased from Sigma-Aldrich (Steinheim, Germany). All other chemicals were of analytical grade.

### 2.2. Preparation of the Spray Freeze-Dried Particles

SFD was conducted with a method slightly modified and adapted from the method described earlier [10,11]. For droplet formation, a monodisperse droplet generator (MTG-01-G1, FMP Technology GmbH, Erlangen, Germany) with a nozzle diameter of 50 or 100 μm was installed on top of the spray tower. For the insulin formulations, a solution of mannitol [5% *w/v*], PVP [1% *w/v*] and insulin [2% *w/v* or 6 IU/mg] with or without sodium taurocholate or β-cyclodextrin [1% *w/v*] was sprayed into the spray tower. Mannitol was used as a cryoprotectant and bulking agent, PVP for mechanical stability, and sodium taurocholate or β-cyclodextrin as a penetration enhancer. The freezing of the droplets took place within the cooled stainless steel spray tower enclosed in a cooling jacket of liquid nitrogen where direct spraying into the liquid nitrogen was avoided. The droplets were frozen at −130 °C and the frozen particles were collected in a cooled container for further freeze drying. Obtained particles were then freeze dried in a Martin Christ Alpha 1-4 LSC plus freeze dryer (Osterode am Harz, Germany). The collected powder contained 21.8% (*w/w*) insulin, 55.8% (*w/w*) mannitol, 11.2% (*w/w*) PVP, and 11.2% (*w/w*) sodium taurocholate or β-cyclodextrin.

For the FITC-labeled dextran formulations, solutions of FITC-labeled dextran (various molecular weights: 4, 10, 20, 40, and 70 kDa; accordingly, these formulations are referred to as FD4 to FD70 in the following text) [6% *w/v*], PVP [1% *w/v*] and sodium taurocholate [1% *w/v*] were sprayed into the spray tower. For fluorescein sodium formulations, a solution of mannitol [5% *w/v*], PVP [1% *w/v*], and fluorescein sodium [0.05% *w/v*] were sprayed into the spray tower.

### 2.3. SFD Particle Characterization

#### 2.3.1. Scanning Electron Microscopy 

A Hitachi SU3500 scanning electron microscope (Hitachi, Tokyo, Japan) was used for the imaging of the SFD particles to examine the surface morphology. SFD samples were mounted on aluminum pins using double adhesive tape and sputter-coated with gold (Polaron SC7640 Sputter Coater, Quorum Technologies Ltd., Newhaven, UK). The samples were placed onto the sample holder of the scanning electron microscope and analyzed.

#### 2.3.2. Confocal Microscope Imaging

The distribution of FITC-labeled insulin in the SFD particles was evaluated using a Nikon Eclipse Ti A1 Laser Scanning Confocal Imaging System (Nikon, Tokyo, Japan) equipped with a modular laser system and an inverted Nikon microscope. Insulin was labeled with fluorescein isothiocyanate (FITC) based on the reaction between the isothiocyanate group of FITC and the amine groups of insulin, as previously described [18,19]. Then, 5 mg of FITC was dissolved in 1 mL of DMSO and added drop by drop into a 20 mL of 0.1 M sodium carbonate solution containing 100 mg of insulin. Upon addition of FITC, the reaction vials were protected from light and mixed under magnetic stirring for 12 h at 4 °C. 7 mL of 0.2 M ammonium chloride solution was added into the reaction vials under magnetic stirring for 2 h to quench the excess FITC. The mixture was then dialyzed (1000 MWCO) and lyophilized to obtain the FITC-labeled insulin powder. SFD particles were prepared with labeled insulin to determine the distribution of insulin in the SFD particles under confocal microscope with the argon-ion-laser line (excitation: 488 nm/Emission: 524 nm).

#### 2.3.3. Particle Size and Distribution

The particle size distribution of the particles was measured by dynamic image analysis using a Camsizer^®^ X2 instrument coupled with X-Dry module with X-Jet (Mictrotrac Retsch, Düsseldorf, Germany). The volume median particle size (d_50_), d_10_, d_90_ and the span values were obtained. The measurements were performed as *n* = 6.

#### 2.3.4. Specific Surface Area

The specific surface area was measured with a BET Quantachrome Nova 3200 high speed gas sorption analyzer (Quantachrome, Boynton Beach, FL, USA). Nitrogen was used as an adsorption gas at temperature of 77 K.

#### 2.3.5. Aerodynamic Properties and Particle Deposition

The aerodynamic properties of the SFD powder formulations were determined using a Next Generation Cascade Impactor (NGI) equipped with a critical flow control unit (Copley Scientific, Nottingham, UK) and 1 L nasal extension chamber set up according to the manufacturer [20] (see also Supplementary Material Figure S1). SFD particle formulations were given through the entry port of the nasal expansion chamber via a custom-made nasal device to simulate the nasal administration with an air flow rate of 15 L/min which is regarded as normal inspiration flow rate [21]. The effective cut-off aerodynamic diameters for each stage at 15 L/min are 14.1, 8.6, 5.4, 3.3, 2.1, and 1.0 µm for stages 1–7, respectively. The amount of insulin remained in the extension chamber and the stages were then determined by HPLC as described under “insulin analytics” and the amount of fluorescein sodium was determined fluorometrically (excitation: 485 nm/emission: 535 nm) using a Plate Reader 1420 Multilabel Counter VICTOR^3^V^®^ (Perkin Elmer, US-Waltham, MA, USA), in order to determine the fraction deposited in the nose and the inhalable fraction. Each sample was analyzed in triplicate and the results were shown as mean ± SD.

#### 2.3.6. Insulin Analytics

Insulin in the SFD particles was quantified by HPLC using a Waters 2695 HPLC System equipped with a 996 photodiode array detector (Waters, Milford, MA, USA) according to the human insulin monograph of the European Pharmacopoeia 9.0. Analysis was carried out with a 250 mm RP-C18 column (LiChrospher^®^ 100 RP-18 5 μm, Merck, Darmstadt, Germany). The flow rate was 1 mL/min, the injection volume was 100 μL, and the UV detection wavelength was 214 nm. Each sample was analyzed in triplicate. Size exclusion chromatography was performed with a Zorbax GF-250 column (Agilent Technologies, Santa Clara, CA, USA), where the mobile phase was 0.2 M pH 7.0 phosphate buffer containing 0.005% sodium azide at a flow rate of 1.0 mL/min. The UV detection wavelength was 275 nm. Each sample was analyzed in triplicate in comparison with a chromatogram of a reference fresh insulin sample.

### 2.4. In Vivo Experiments

All animal experiments were performed in accordance with the EU Directive 2010/63/EU on the protection of animals used for scientific purposes. In vivo experiments were run at the animal facility of the University of Burgundy/Franche-Comté (Besançon, France) in compliance with the French legislation on animal experimentation under the Project ‘Exp An N2 EA4267 2015-2020’, previously accepted by the ethical committee CEBEA 58. Then, 200 g Sprague Dawley albino male rats were acclimatized to laboratory conditions for one week in a ventilated room at 22 °C and 45% relative humidity with a 12:12 h light/dark cycle, before the beginning of the experiments. Rats were given access to food and water *ad libitum*.

Blood glucose levels after the administration of insulin loaded SFD particles were tested as follows: Rats were prevented from access to food one hour prior to the start of the experiments and kept in fasting conditions with free access to water during the duration of the experiments. SFD particle formulations were administered nasally via a custom-made nasal device (Supplementary Material Figure S5) through both nostrils under anesthesia with isoflurane. SFD formulations with sodium taurocholate or β-cyclodextrin, the formulation without any penetration enhancer [30 IU/kg] and isotonic saline solution [50 μL] were administered nasally. Insulin reference in isotonic saline solution was administered intravenously with an injection into the superficial dorsal vein of the penis [1 IU/kg] after sterile filtration. Blood samples were taken from the caudal vein in predetermined time intervals (0, 2, 10, 20, 40, 60, 90, 120, and 180 min after administration) and the glycemic effect of the formulations was measured by a blood glucose meter (Glucofix^®^ Sensor, A. Menarini Diagnostics, Florence, Italy). The area above the curve (AAC) of the concentration versus time profile was calculated with the linear trapezoidal method. The insulin availability (F) of the SFD particles with various penetration enhancers was calculated based on the pharmacological effect as F = (AAC_nasal_/dose_nasal_)/(AAC_iv_/dose_iv_) × 100.

The currently observed initial increase in blood glucose level in all groups, which was related to the stress of the administration [22], was included in the calculations since this was not significantly different for all groups.

FITC-labeled dextran SFD formulations with and without sodium taurocholate (4 kDa) and sodium taurocholate containing SFD formulations with FITC-labeled dextran (10, 20, 40, 70 kDa) [7.5 mg/kg] were tested for their nasal bioavailability by an equivalent procedure. SFD particle formulations were administered nasally via the same nasal device through both nostrils under anesthesia with isoflurane. Reference solutions of each formulation were administered intravenously with an injection into the superficial dorsal vein of the penis [1 mg/kg] after sterile filtration. Thereafter, blood samples were taken at the equivalent time points from the caudal vein (0, 2, 10, 20, 40, 60, 90, 120, and 180 min after administration), the plasma was then separated from the blood samples by centrifugation and consequently, the amount of FITC-labeled dextran was quantified in plasma samples fluorometrically. Again, the area under the curve (AUC) of the concentration versus time profile was calculated with the linear trapezoidal method. The nasal bioavailability (F) of FITC-labeled dextrans of various molecular weights was calculated as F = (AUC_nasal_/dose_nasal_)/(AUC_iv_/dose_iv_) × 100. 

### 2.5. Statistical Analysis

Statistical analysis was conducted using Sigmastat 4.0 software (Systat Software, Inc., San Jose, CA, USA) and Graphpad Prism 8 (GraphPad Software, Inc., San Diego, CA, USA). Statistical difference was determined using ordinary one-way ANOVA and followed by multiple comparisons using Dunnett’s test. The data were expressed as mean ± SD and treatments were considered significantly different if *p* < 0.05.

## 3. Results

### 3.1. Physicochemical Characteristics of Spray Freeze-Dried Particles

**Figure 1:**
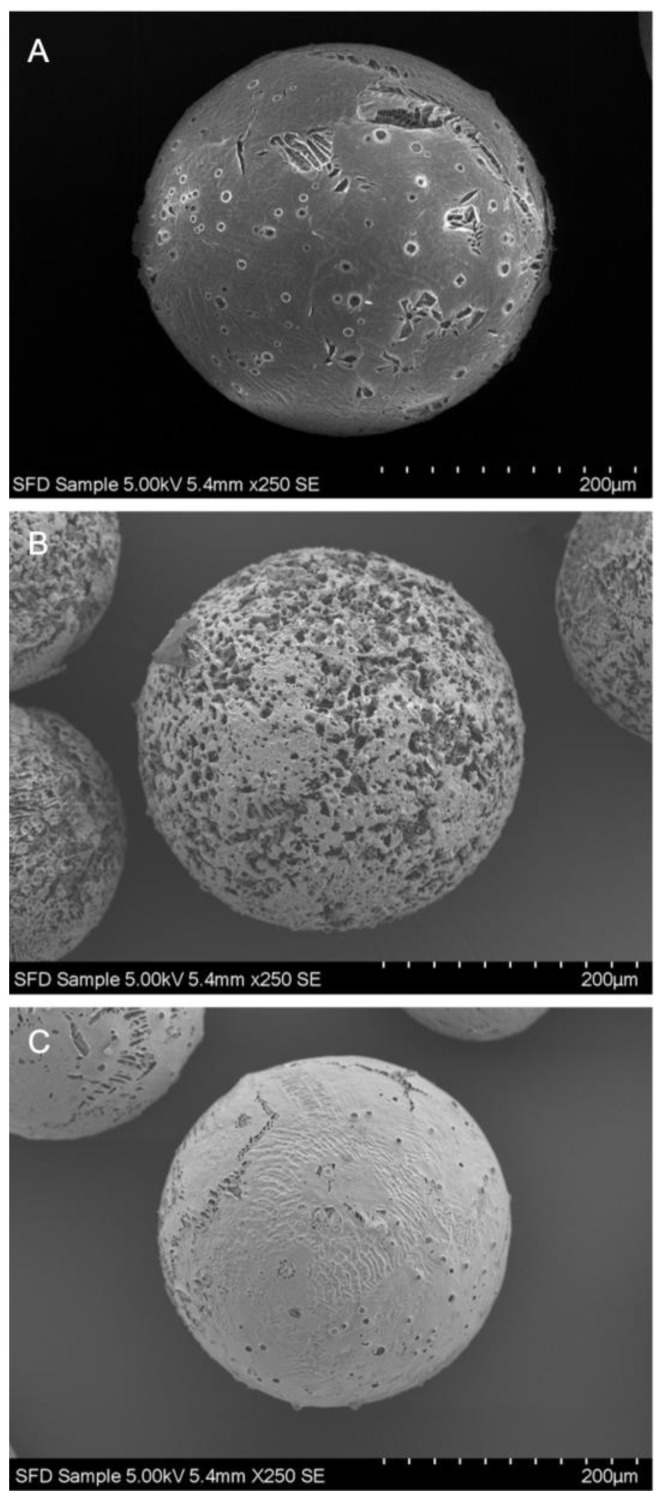
SEM images of insulin loaded SFD particles without penetration enhancer (**A**), with sodium taurocholate (**B**), or with β-cyclodextrin (**C**).

**Figure 2:**
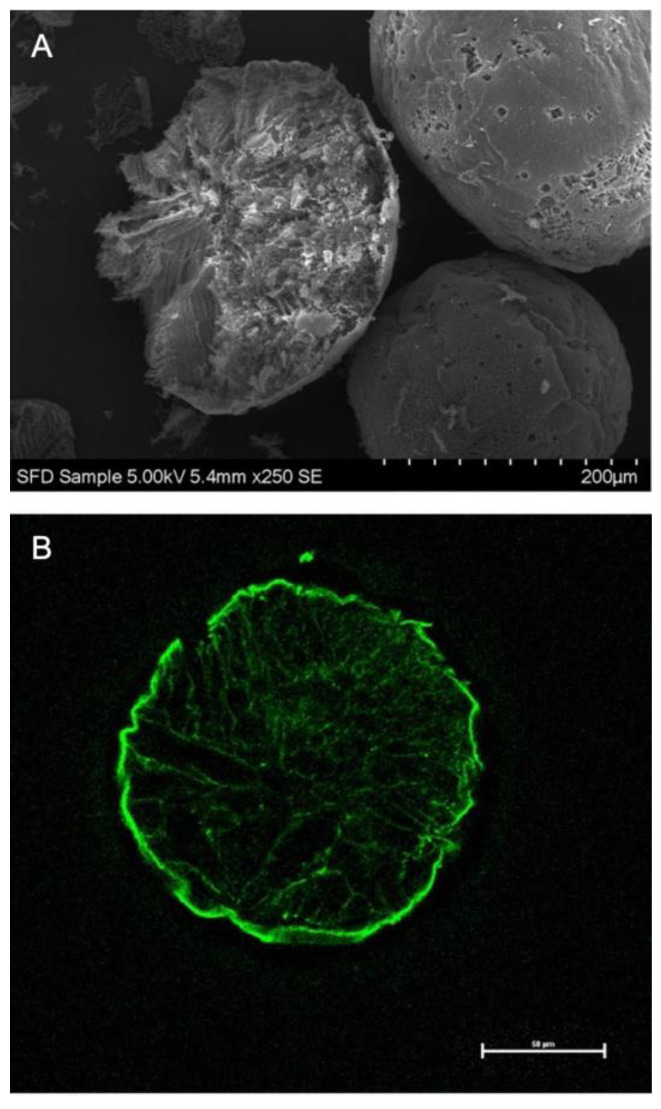
Cross-sections of insulin loaded SFD particles without any penetration enhancer imaged by SEM (**A**) or confocal laser scanning microscopy (**B**) containing FITC-labeled insulin.

SFD particles were spherical and porous throughout all batches. The use of different penetration enhancers resulted in different surface morphologies, however the general shape remained equivalent and only minor differences were observed in view of the lamellae formation of the porous structure (Figure 1). Confocal microscopy of FITC-labeled insulin SFDs revealed a homogenous distribution of insulin throughout the SFD matrix along the lamellae and confirmed the same porous structural entities that were observed in SEM cross-sections (Figure 2). The specific surface area of the SFD formulations was ranging between 7.9 ± 0.5 and 11.8 ± 0.4 m^2^/g. The residual water content in the final SFD formulation was below 2.1% directly after freeze-drying, and despite the large surface area, only minor moisture absorption was observed during storage at −20 °C over 6 months with moisture levels not exceeding 2.5% (Supplementary Material Table S1 and Figure S3).

**Figure 3:**
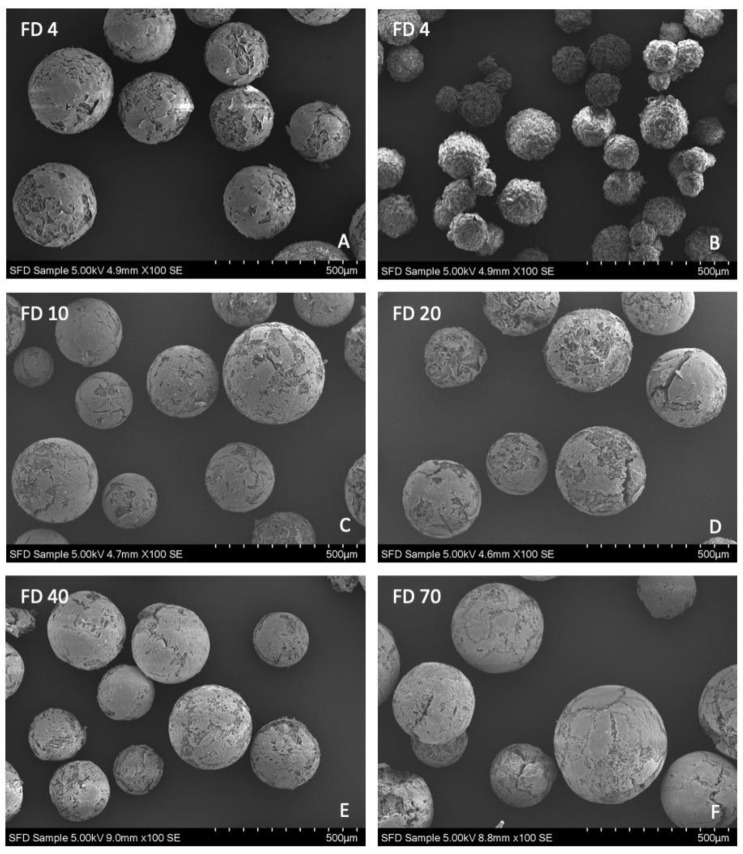
SEM images of FITC-dextran loaded SFD particles showing FD 4 without penetration enhancer (**A**), FD 4, FD 10, FD 20, FD 40, and FD 70 with sodium taurocholate (**B**–**F**).

**Figure 4:**
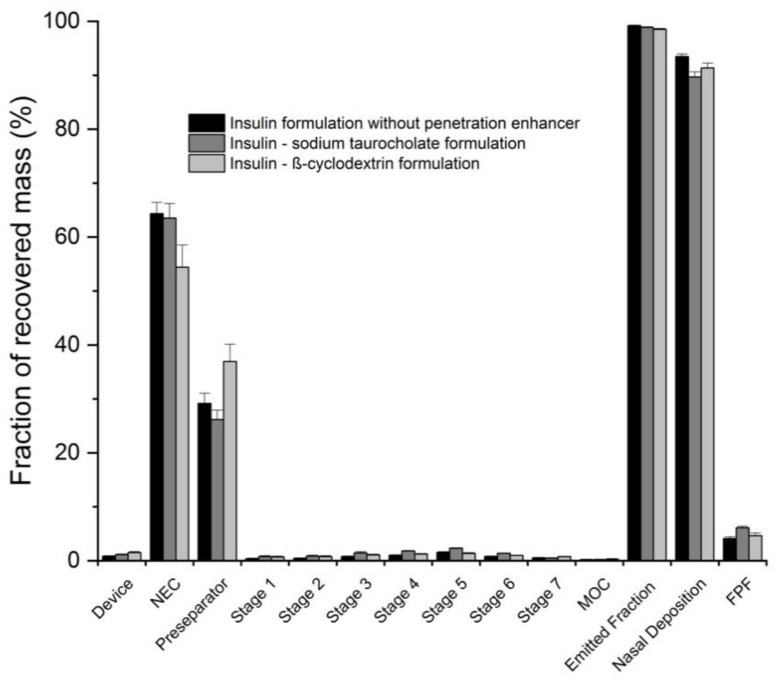
Results of the NGI experiments analyzing the nasal deposition of the insulin loaded SFD particles with different penetration enhancers (*n* = 3).

Particle size distribution data of insulin loaded SFD particles ranged between 208.0 ± 6.0 µm and 249.0 ± 105.2 µm irrespectively whether formulations contained the penetration enhancers sodium taurocholate and β-cyclodextrin or not (Table 1). Similarly, particles loaded with FITC-labeled dextrans led to a similar median diameter range between 190.4 ± 2.4 µm and 222.6 ± 2.6 µm (Figure 3). NGI experiments with a nasal extension chamber, performed in order to confirm the particle size range to be suitable to nasal deposition, revealed a nasal deposition of >90% of the emitted dose which was in turn ranging from 98.5 ± 0.2% to 99.2 ± 0.1 (Figure 4). The fine particle fraction was around 5% with extremes of 4.1 ± 0.3% and 6.1 ± 0.3%, which were found in the insulin formulations (Table 2).

The SEC measurements revealed absence of aggregate or fragment peaks which showed that insulin is present after redissolving of the formulations as a monomer, and comparable insulin content was quantified by the pharmacopoeia RP-HPLC method (Table 3). SEC analysis further showed excellent stability of insulin in all formulation during storage over 6 months at −20 °C (Supplementary Material Table S2).

### 3.2. In Vivo Experiments

#### 3.2.1. In Vivo Testing of Insulin Loaded Particles

**Figure 5:**
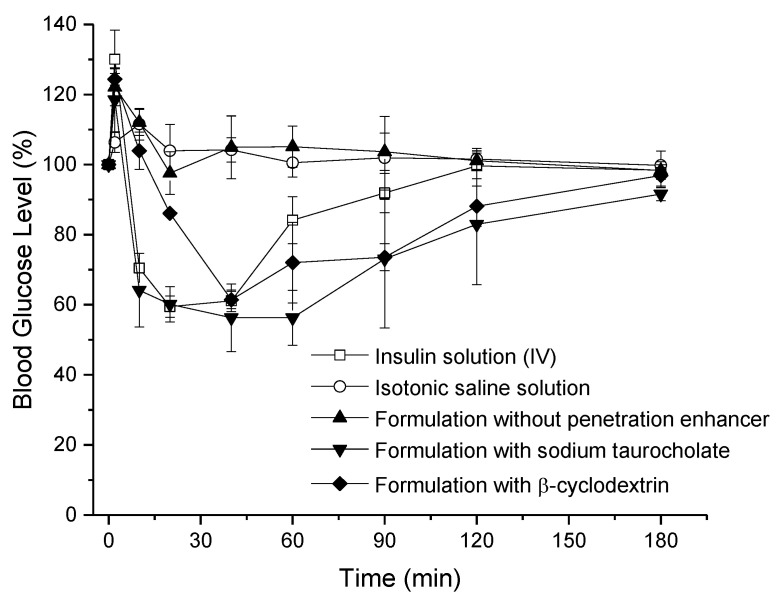
Effect of insulin loaded SFD particles with or without penetration enhancers after nasal administration in rats.

Controls of saline solution did not lower the blood glucose level of the animals, while glucose levels dropped to the minimum after 20 min and recovered quickly after that point for the intravenous control group. Similar to saline controls, nasal insulin SFD without penetration enhancer did not fall below the baseline level which corresponds to levels measured prior to the administration (Figure 5). Hence, the nasal bioavailability of the formulation without any penetration enhancer was not significantly different from zero. However, the addition of penetration enhancers led to significant bioavailability with the most effective formulation containing sodium taurocholate with a nasal bioavailability of 7.0 ± 2.8%, followed by the formulation with β-cyclodextrin with a bioavailability of 4.4 ± 0.7% (Table 4). Similar to the IV reference group, nasal insulin formulations with penetration enhancers led to a sharp decrease after administration, maintained the lowered level for a longer period, and returned to the baseline gradually. The formulation with β-cyclodextrin was able to decrease the blood glucose level considerably after the small increase after the administration, although it was not as low as sodium taurocholate formulation. 

#### 3.2.2. In Vivo Testing of FITC-Labeled Dextran Containing SFD Particles

**Figure 6:**
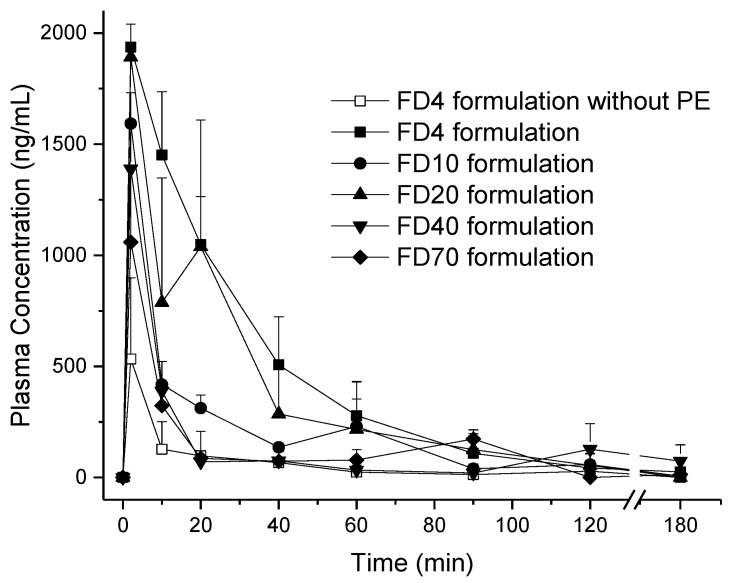
Plasma concentration versus time profiles of SFD particles loaded with FITC-labeled dextrans of different molecular weights with or without sodium taurocholate as a penetration enhancer after nasal administration in rats (*n* = 3).

**Figure 7:**
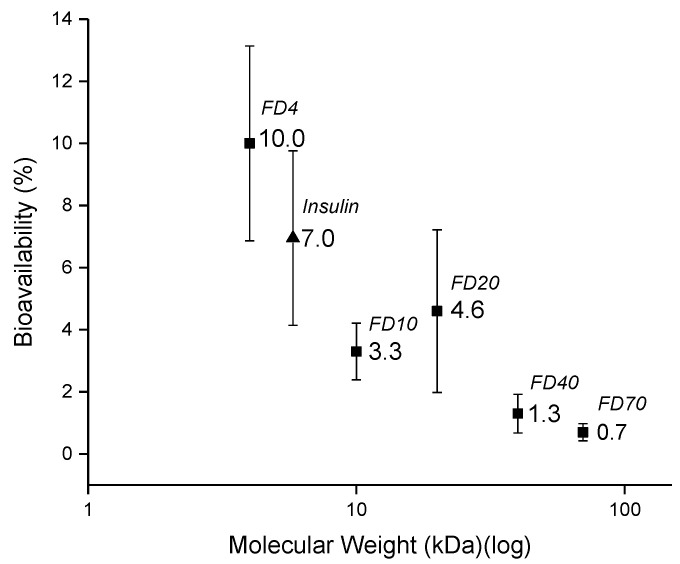
Comparison of the nasal bioavailabilities of FITC-labeled dextrans with different molecular weights and fitting insulin into the trend obtained with dextrans.

All formulations reached maximum concentrations 2 min after administration (Figure 6). FD 4 formulations without penetration enhancer was found to have 1.5 ± 1.3% nasal bioavailability, which increased more than 6-fold to 10.0 ± 3.1% for the FD 4 formulation with sodium taurocholate (Table 5). When integrating constantly the sodium taurocholate in the SFDs, nasal bioavailability was 10.0 ± 3.1% for FD 4 with sodium taurocholate, gradually decreasing towards 0.7 ± 0.3% for FD 70. It was found that the nasal bioavailability of the various FITC-dextrans decreased with their increasing molecular weight. The subsequent plot of FITC-dextran bioavailabilities versus their respective molecular weights underlined this tendency (Figure 7). 

## 4. Discussion

When combining freeze drying and spray drying to SFD, the resulting particles hold several unique properties and advantages over one of the two techniques alone. Lyospheres are spherical in shape and possess an adjustable low density, based on the solid content that is initially inserted in the formulation. In principle, their internal morphologies show strong similarities to traditional freeze-drying cakes, however, due to the spherical shape and a narrow particle size distribution, lyospheres possess a very good powder flowability. In a previous publication from our research group, Eggerstedt et al. [10] found the apparent density of the SFD microspheres to be in a range between 0.06 and 0.36 g/cm^3^, while the formulations with a solid content similar to the formulations in this study had an apparent density between 0.1–0.2 g/cm^3^. In the same study, the porosity was calculated to be ranging between 73.2 and 95.8% for SFD particles with varying solid content. The formulations with a similar solid content to the present study were found to have porosity between 90–95%. The large porosity and surface area as determined by BET strongly contribute to the rapid dissolution. 

The efficient nasal targeting with low pulmonary exposure can be effectively controlled by the formulation composition and the SFD-process. With a higher concentration of excipients, a higher viscosity of the initial spraying solution and a slightly increased particle size can be observed and this in parallel can augment the aerodynamic diameter of the particles, ensuring nasal instead of pulmonary deposition. In contrary, reducing the density, which allows for less material input by maintaining a constant particle size, is bearing the risk of reduced mechanical particle stability, leading to fragments that still can reach pulmonary tissues, due to the subsequently lower aerodynamic diameter, which also became visible in the current study with about 5% of the particles deposited beyond the nasal cavity. 

Typically, freezing risks are a major destabilizing stress on protein and peptide drugs during freeze drying. The continuously progressing phase separation into ice, and a highly concentrated viscous liquid composed of the active cryoprotectants and other components of the formulation, can lead to protein aggregation [23]. Moreover, the crystallization of ice may exert an additional mechanical stress on the active, which may result in its aggregation. When producing SFD, the liquid stream of fine droplets exhibits a high surface to volume ratio which in turn facilitates efficient and highly accelerated freezing of the droplets (a few milliseconds) compared to classical freeze-drying procedures. 

The stabilizing mechanisms of cryoprotectants are complex and not yet entirely understood [24] however, as shown in precedent studies, SFD was observed to be much more robust against changes, probably due to the accelerated freezing step [11]. With SFD, a rather generic choice of cryoprotectant and concentration could be possible, which can very much simplify the development process of the lyophilizate formulation. Within the SFD process, shear stress at drop break-up is the main remaining stress factor, however, the unchanged activity of insulin confirmed the suitability of the process. Similar findings were reported earlier for other peptides and proteins, e.g., lysozyme and bovine serum albumin [10].

The strongly enlarged surface of the lyospheres, together with their relatively small size, also allows for faster drying in contrast to the bulk-frozen product which can accelerate the drying step in general. Finally, producing uniformly sized particles is also favorable for a homogeneous drying process, as only the small radius of the particles (of few hundred microns) represents the layer that water molecules need to overcome during the drying step which is much smaller than the one in classical freeze-drying vials. 

Among other biological barrier-related physiological mechanisms, nasal mucosa gradually limits the permeation for drug molecules simply due to an increasing molecular weight [25] and a similar trend was observed in our in vivo experiments with a range of dextrans of increasing molecular weights. 

Various bile salts and derivatives, cyclodextrins, chitosans, synthetic and natural surfactants have been tested as penetration enhancers for the nasal delivery and have been found effective in varying degrees [4,26,27,28]. 

First attempts for intranasal delivery with porcine and bovine insulins were solution-based and administered as sprays [29,30]. Thereafter, alternative spray-based formulations showed significant nasal bioavailability in men (about 15%) with an onset of action after 10 to 15 min [31,32], which is a comparable outcome to our study. 

With the aid of the penetration enhancers, the in vivo bioavailability of insulin increased several folds. This result is parallel to the findings of earlier in vivo studies and clinical trials. Nasal bioavailability of insulin without penetration enhancers was found to be about 1–2%, and increased to 5–20% when a range of penetration enhancers were added to the formulations [33,34,35]. Compared to oral administration with bioavailability of less than 1%, the nasal bioavailability of 7% achieved with lyospheres indicates a considerable amount of drug absorbed considering its large molecular weight (5.8 kDa) and dry formulation. 

The predictability of the in vivo results obtained in rats to the performance of the lyospheres in humans is limited given by the differences in anatomy, size, and cellular biochemistry [36,37]. Furthermore, conscious administration with synchronized inspiration may further enhance the nasal deposition and bioavailability in humans in comparison to involuntary administration to rats. 

One factor that needs to be kept in mind is the content of the permeation enhancers in the precedent studies as well as in this paper. Bile salts, fatty acid derivatives, or surfactants increased the mucosal permeability of insulin but they concomitantly increase the risks for local irritation, nasal secretion, sneezing, or burning sensation [31,32]. Additionally, the presence of surface-active components has been reported to impede the structural integrity of SFD particles [38].

More recent approaches intend to use nasal insulin in a different therapeutic context, which is the nose-to-brain delivery in cognitive impairment [39,40]. Although, in these studies, the focus was mainly on the permeation modifying components, such as nanocarrier-assisted delivery, rather than on the question of a liquid or a solid final dosage form, this also underlines the general benefit of a freeze-dried particle system for nasal drug delivery. 

## 5. Conclusions

Lyospheres allowed for successful administration of insulin via the nasal route in rats. Our findings in terms of the permeation enhancing effect were not dramatically different from precedent studies integrating such excipients into the nasal formulation. From the biopharmaceutical perspective, SFD does not alter general behavior of these excipients, which further underlines the feasibility of lyospheres for nasal administration as an alternative to nasal sprays. The SFD process proved to maintain the biological activity of insulin and hence may provide a useful platform to formulate macromolecules maintaining their integrity and therapeutic activity and is seemingly a very promising formulation approach for nasal administration of macromolecules.

## Figures and Tables

**Table 1:** Particle size distribution of insulin or FITC-dextrans loaded SFD particles.

SFD Formulation	Median Diameter ± SD (µm)	SPAN ± SD
w/o penetration enhancer	208.0 ± 6.0	0.5 ± 0.03
+ sodium taurocholate	249.0 ± 15.2	0.6 ± 0.3
+ β-cyclodextrin	209.0 ± 2.3	0.5 ± 0.01
FD 4 w/o PE	209.5 ± 8.6	1.5 ± 0.01
FD 4	200.1 ± 0.6	1.1 ± 0.2
FD 10	222.6 ± 2.6	0.9 ± 0.02
FD 20	190.4 ± 2.4	1.2 ± 0.05
FD 40	221.2 ± 2.9	0.9 ± 0.02
FD 70	213.9 ± 5.0	0.8 ± 0.01

**Table 2:** NGI data for the insulin formulations.

Insulin Formulation	Emitted Fraction (%)	Nasal Fraction (%)	Nasal Fraction as % of the Emitted Dose	FPF (%)
Formulation without penetration enhancer	99.2 ± 0.1	93.5 ± 0.5	94.3 ± 0.5	4.1 ± 0.3
Formulation with Sodium taurocholate	98.9 ± 0.1	89.7 ± 1.0	90.7 ± 1.0	6.1 ± 0.3
Formulation with β-cyclodextrin	98.5 ± 0.2	91.3 ± 0.9	92.7 ± 0.9	4.7 ± 0.5

**Table 3:** Percentage of the theoretical insulin content determined by RP-HPLC and SEC.

Insulin Formulation	RP-HPLC (%)	SEC (%)
w/o penetration enhancer	97.6 ± 0.8	98.1 ± 1.3
+ sodium taurocholate	90.4 ± 0.2	91.9 ± 3.3
+ β-cyclodextrin	97.1 ± 2.6	98.7 ± 2.6

**Table 4:** Pharmacokinetic parameters obtained from the in vivo trials on rats.

Insulin Formulation	Insulin Dose (IU/kg)	C_min_ (%)	T_min_ (min)	AAC (% min)	F (%)
Insulin solution (IV)	1	59 ± 3	20	2234 ± 579	-
w/o penetration enhancer (nasal)	30	98 ± 6	20	−531 ± 577	−0.8 ± 0.9
+ sodium taurocholate (nasal)	30	56 ± 10	40	4659 ± 1886	7.0 ± 2.8 *
+ β-cyclodextrin (nasal)	30	61 ± 2	40	2940 ± 468	4.4 ± 0.7 *

* *p* < 0.05 compared to SFD w/o penetration enhancer, no significant differences between + sodium taurocholate and + ß-cyclodextrin.

**Table 5:** Pharmacokinetic parameters obtained for the various FITC-dextrans loaded SFDs.

FD Formulation	FD Dose (mg/kg)	C_max_ (ng/mL)	T_max_ (min)	AUC (min·ng/mL)	F (%)
FD 4 kDa solution (IV)	1	7483 ± 2474	0	81,834 ± 24,418	-
FD 4 kDa Formulation w/o PE (Nasal)	7.5	533 ± 366	2	8891 ± 7880	1.5 ± 1.3
FD 4 kDa Formulation (Nasal)	7.5	1938 ± 102	2	61,495 ± 19,258	10.0 ± 3.1 *
FD 10 kDa solution (IV)	1	10,639 ± 2818	0	115,023 ± 20,320	-
FD 10 kDa Formulation (Nasal)	7.5	1593 ± 139	2	28,869 ± 7880	3.4 ± 1.0
FD 20 kDa solution (IV)	1	14,536 ± 3256	0	144,103 ± 30,065	-
FD 20 kDa Formulation (Nasal)	7.5	1892 ± 4	2	49,481 ± 28,330	4.6 ± 2.6
FD 40 kDa solution (IV)	1	13,477 ± 2456	0	236,224 ± 75,785	-
FD 40 kDa Formulation (Nasal)	7.5	1388 ± 207	2	22,367 ± 11,008	1.3 ± 0.6
FD 70 kDa solution (IV)	1	10,934 ± 3194	0	369,050 ± 69,265	-
FD 70 kDa Formulation (Nasal)	7.5	1059 ± 342	2	18,521 ± 7695	0.7 ± 0.3

* *p* < 0.05 compared to SFD w/o penetration enhancer, no significant difference between FD 10, 20, 40, and 70 SFDs compared to the FD4 formulation w/o PE.

## Supplementary Materials

The following are available online at https://www.mdpi.com/article/10.3390/pharmaceutics13060852/s1, Figure S1: Experimental setup of NGI with nasal extension chamber; Figure S2: Custom made device for nasal administration of the particles to rats; Figure S3: Residual moisture content of the formulations after production, and 1, 3 and 6 months after SFD; Figure S4: The particle size distribution diagram of SFD particles with insulin and sodium taurocholate measured after insufflation through the device by dynamic image analysis using a Camsizer X2 instrument (*N* = 3); Figure S5: Images of the intact spherical SFD particles (above) and fractured pieces (below) after insufflation through the device captured by the camera of the Camsizer X2 instrument; Table S1: TG analysis results of the formulations kept at −20 °C 1, 3 and 6 months after SFD (*n* = 3); Table S2: SEC analysis results of the formulations kept at −20 °C 1, 3 and 6 months after SFD (*n* = 3).
