# Supplementary material for: Spray Freeze Dried Lyospheres® for Nasal Administration of Insulin
Source: Pharmaceutics. 2021 Jun 8;13(6):852. doi: 10.3390/pharmaceutics13060852 (PMC8229095; doi:10.3390/pharmaceutics13060852)
Supplement: Supplementary file 1 [file pharmaceutics-13-00852-s001.zip › pharmaceutics-1211971-supplementary.pdf]

pharmaceutics

MDPI

# Supplementary Materials: Spray Freeze Dried Lyospheres® for Nasal Administration of Insulin

**Tugrul Mert Serim, Jan Kožák, Annika Rautenberg, Ayse Nurten Özdemir, Yann Pellequer and Alf Lamprecht**

## NGI Setup with Nasal Extension Chamber

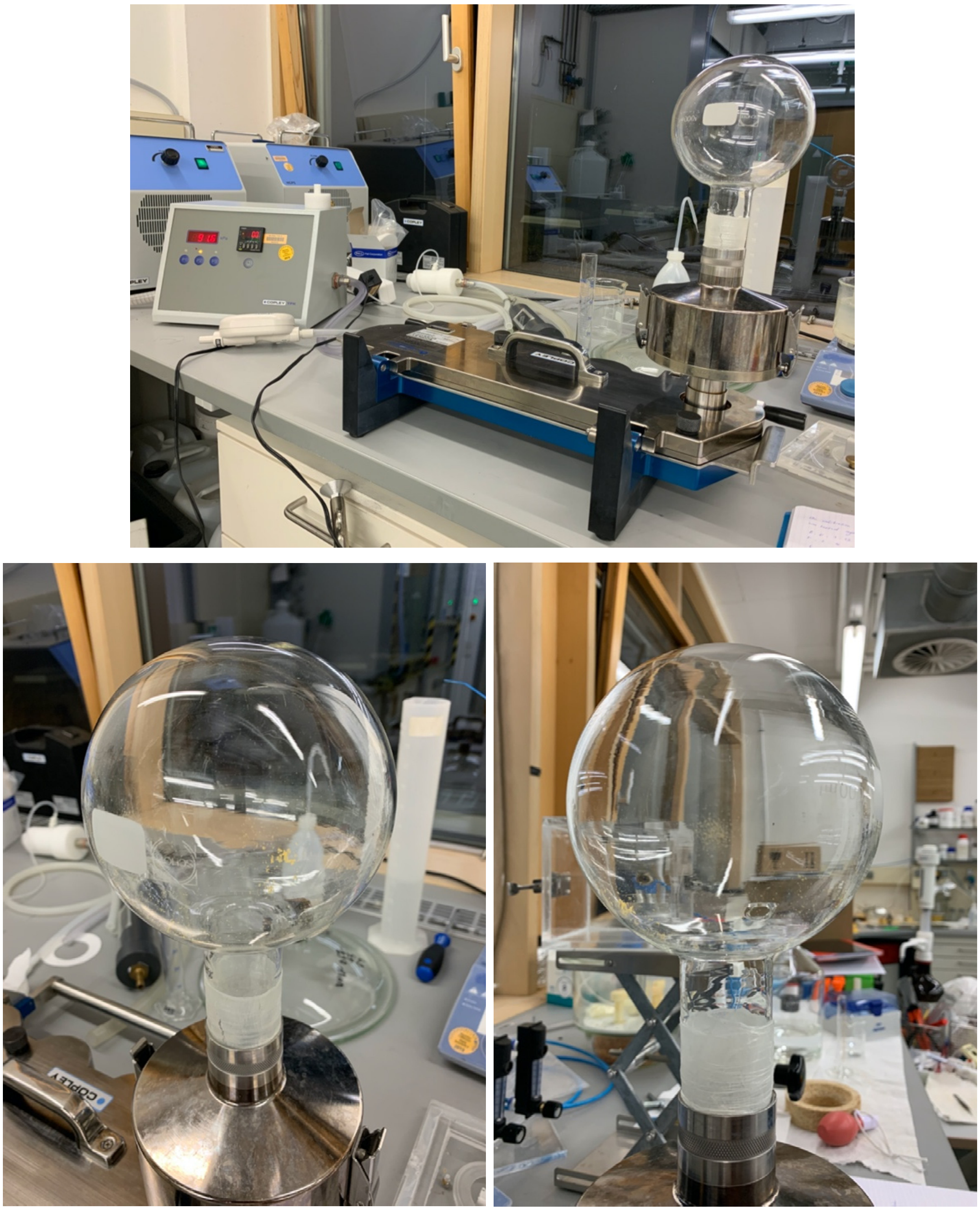

**Figure S1.** Experimental setup of NGI with nasal extension chamber.

### Custom Made Device for Nasal Administration

A custom-made device is an insufflation device made up of a glass tube integrated with a blunt-end needle and a hand pump. Needles of different gauges could be used depending on the particle size of the formulations. The hand pump allows single-hand use, which facilitates the application to the rat nose, while holding the rat with the other hand. The device offered flexibility during the in vivo experiments.

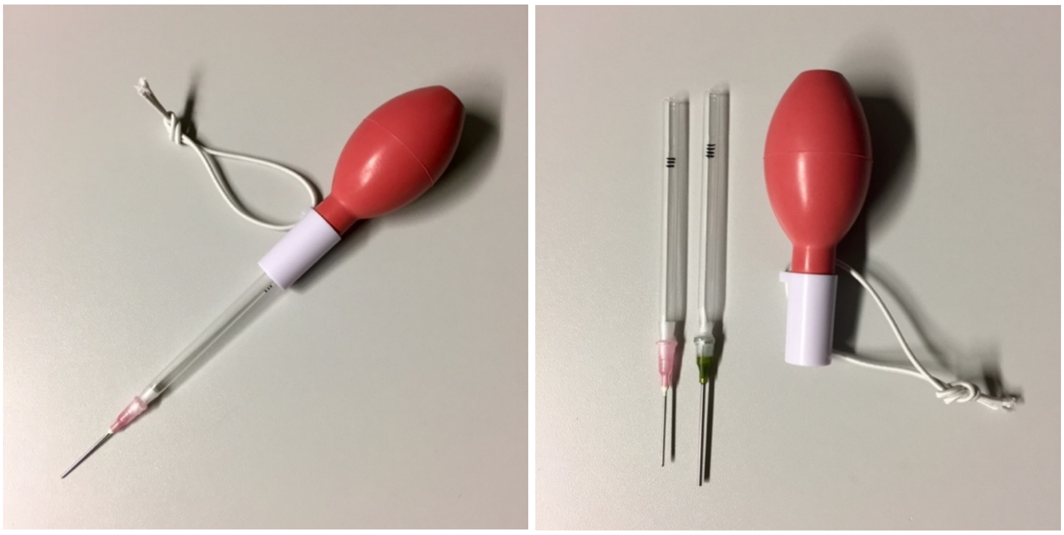

**Figure S2.** Custom made device for nasal administration of the particles to rats.

### Residual Water Content and Moisture Uptake

The formulations were kept at -20°C, and the residual moisture content was measured gravimetrically with a thermogravimetric (TG) analyzer (PerkinElmer TGA 7 Thermogravimetric Analyzer) after 1, 3 and 6 months. The water content did not exceed 2.5 % (w/w) in any of the formulations. Although porosity might cause higher moisture uptake levels, moisture content of the SFD did not drastically increase in 6 months of measurement. The powder formulations were freely flowing and no sticking or agglomeration was observed in the defined time points.

**Table S1.** TG analysis results of the formulations kept at −20 °C 1, 3 and 6 months after SFD ($n$ = 3).

| Insulin Formulation | After SFD | Loss on Drying (%) | | |
|---|---|---|---|---|
| | | 1 Month | 3 Months | 6 Months |
| without penetration enhancer | 1.4 ± 0.04 | 1.5 ± 0.1 | 1.8 ± 0.1 | 1.8 ± 0.2 |
| with Sodium taurocholate | 2.1 ± 0.2 | 2.1 ± 0.1 | 2.3 ± 0.1 | 2.3 ± 0.1 |
| with β-cyclodextrin | 2.0 ± 0.1 | 2.2 ± 0.1 | 2.4 ± 0.1 | 2.5 ± 0.2 |

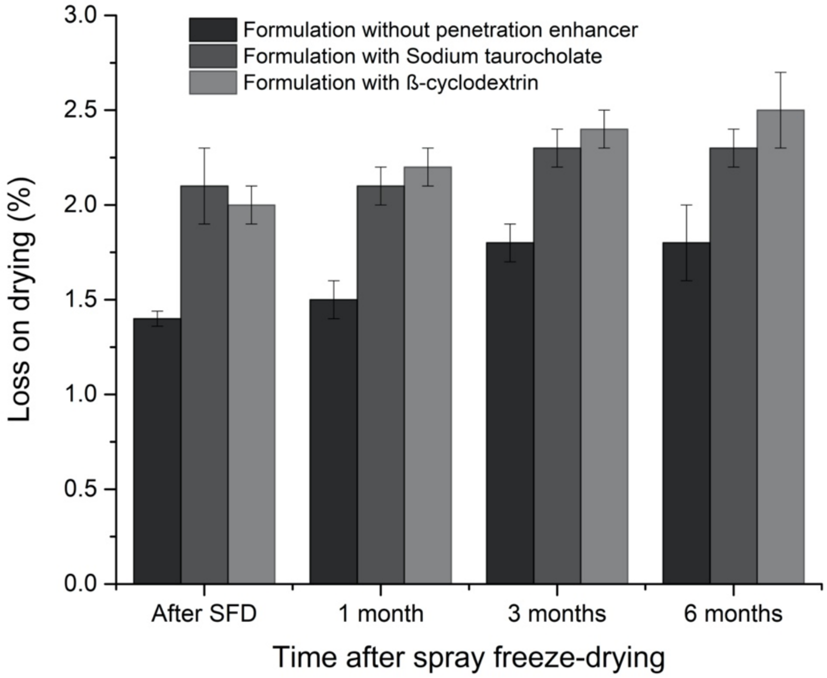


**Figure S3.** Residual moisture content of the formulations after production, and 1, 3 and 6 months after SFD.

## Long-term Stability

SEC analysis after 1, 3 and 6 months revealed similar results to the SEC analysis right after the production of the particles, which showed that insulin remained stable during the time it was tested. There was a single peak for insulin corresponding to the one in the reference chromatogram, the area of the chromatogram was comparable to the reference and no other peak due to degradation or aggregation was observed for 6 months.

**Table S2.** SEC analysis results of the formulations kept at −20 °C 1, 3 and 6 months after SFD (*n* = 3).

| Insulin Formulation | Amount of Insulin (%) after Production * | | |
|---|---|---|---|
| | 1 Month | 3 Months | 6 Months |
| without penetration enhancer | 100.1 ± 0.2 | 100.3 ± 0.5 | 99.8 ± 0.1 |
| with Sodium taurocholate | 100.4 ± 0.6 | 99.5 ± 0.3 | 100.2 ± 0.3 |
| with β-cyclodextrin | 99.1 ± 0.5 | 99.0 ± 0.6 | 99.3 ± 0.5 |

*Amount of insulin in the formulations 1, 3 and 6 months after SFD as percentage of the amount measured at the beginning

## Particle Size and Morphology

The particle size distribution of the particles was measured after insufflation through the device by dynamic image analysis using a Camsizer X2 instrument. Particle size distribution diagram of SFD particles with insulin and sodium taurocholate revealed that there is a fraction with a very small size, which could be the fragments ruptured due to the impact of the insufflation. This could also explain 6.1 ± 0.3 % FPF found in this formulation with the NGI. Camsizer X2 instrument records every particle passing in front of its camera. A compilation of the images of the intact particles and fragments can be seen in Figure S6.

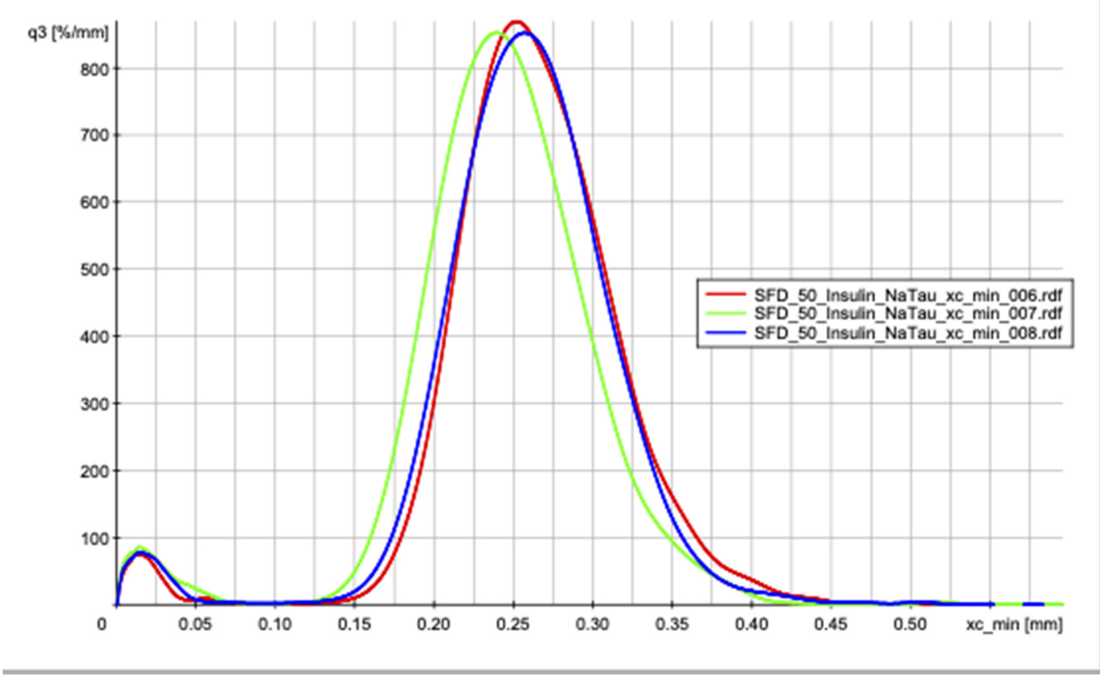


**Figure S4.** The particle size distribution diagram of SFD particles with insulin and sodium taurocholate measured after insufflation through the device by dynamic image analysis using a Camsizer X2 instrument ($N = 3$).

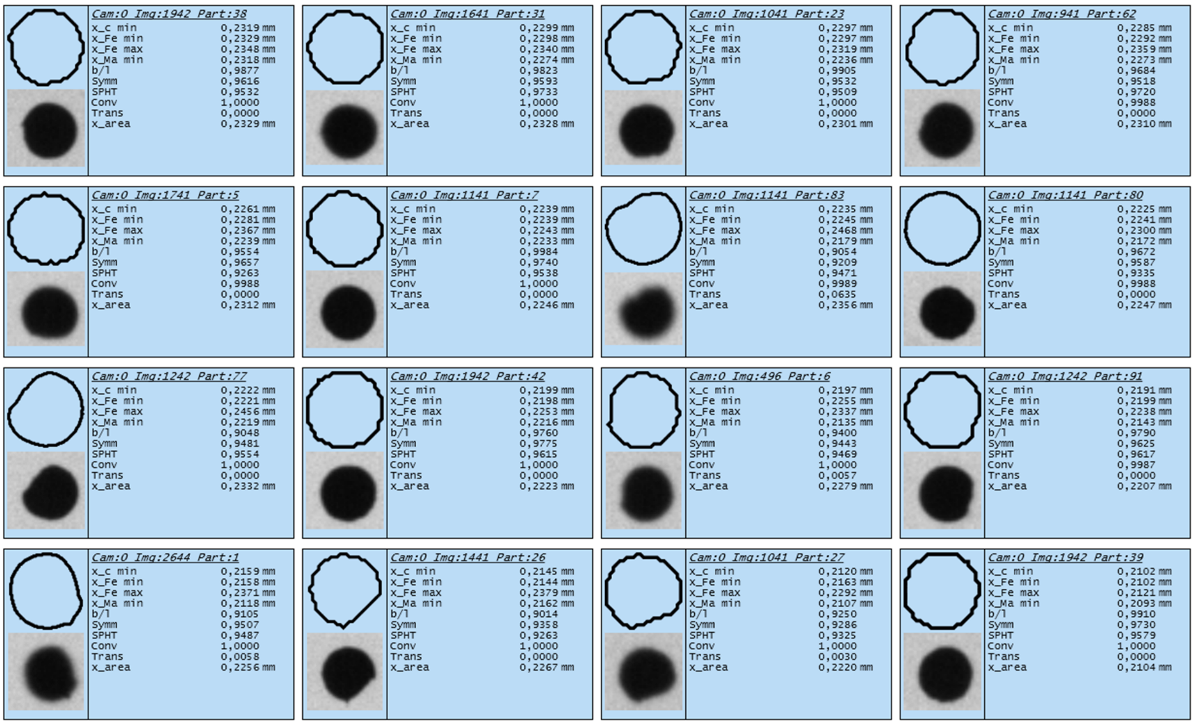

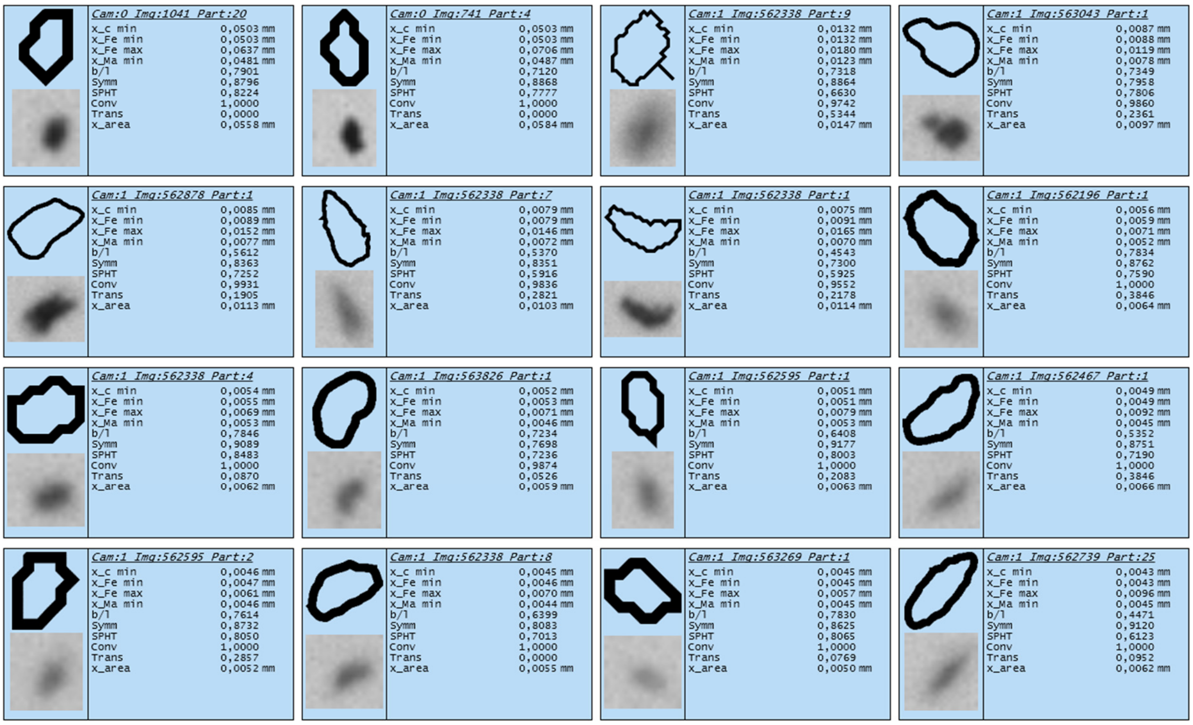


**Figure S5.** Images of the intact spherical SFD particles (above) and fractured pieces (below) after insufflation through the device captured by the camera of the Camsizer X2 instrument.
